# Effects of Chronic Electroacupuncture on Depression- and Anxiety-Like Behaviors in Rats with Chronic Neuropathic Pain

**DOI:** 10.1155/2014/158987

**Published:** 2014-03-26

**Authors:** Qian Li, Na Yue, Shen-Bin Liu, Zhi-Fu Wang, Wen-Li Mi, Jian-Wei Jiang, Gen-Cheng Wu, Jin Yu, Yan-Qing Wang

**Affiliations:** Department of Integrative Medicine and Neurobiology, Institute of Acupuncture Research, School of Basic Medical Sciences, State Key Laboratory of Medical Neurobiology, Institutes of Brain Science, Fudan University, 138 Yi-Xue-Yuan Road, Shanghai 200032, China

## Abstract

Growing evidence indicates that chronic neuropathic pain is frequently accompanied by an array of psychiatric diseases, such as depression and anxiety. Electroacupuncture (EA), as one therapy of traditional Chinese medicine, has displayed potent antidepressant-like effects in numerous clinical studies. The present study was designed to examine the possible effects of EA on the depressive and anxiety disorders induced by neuropathic pain. A classic rat model of neuropathic pain was produced by chronic constriction injury (CCI) of the sciatic nerve. EA was performed on acupoints “Bai-Hui” (GV20) and unilateral “Yang-Ling-Quan” (GB34). The antidepressive and anxiolytic effects of EA treatment were analyzed using the forced swimming test (FST) and the elevated plus maze (EPM) test, respectively. CCI resulted in remarkable depression- and anxiety-like behaviors, whereas the chronic EA treatment significantly improved the behavioral deficits of CCI rats. Moreover, the phosphorylation level of the NMDA receptor type 1 (NR1) subunit was decreased in the hippocampus of CCI rats. Intriguingly, continuous EA treatment effectively blocked this decrease in the levels of pNR1. These results suggested that EA has antidepressive and anxiolytic effects on rats with neuropathic pain and that this might be associated with restoring the phosphorylation of NR1 in the hippocampus.

## 1. Introduction

Chronic neuropathic pain as a persistent stressor induces biochemical, physiological, and psychological changes, which then result in multiple neuropsychiatric disorders [1, 2]. Patients with chronic pain frequently suffer from mental illnesses, particularly depression and anxiety [3]. Because currently available antidepressant treatment has limitations including side effects and the delayed onset of therapeutic efficacy, it is crucial to look for alternative approaches to optimize the treatment [4]. One therapy from traditional Chinese medicine, electroacupuncture (EA), has been widely proved to be effective in treating chronic pain [5, 6]. However, whether continuous EA application has an effect on the depression and anxiety induced by neuropathic pain remains unknown. Therefore, clarifying the possible effects of EA treatment on pain-related psychiatric disorders and revealing its underlying mechanisms may be of clinical benefit.

As one essential subunit of the ionotropic glutamate receptor NMDAR (N-methyl-D-aspartate receptor), NR1 plays a pivotal role in psychiatric disorders [7]. Mice with either knockdown or phosphorylation blockage of NR1 develop obvious negative behaviors, such as reduced social interactions and self-injury [8, 9]. Previous studies also reported that olfactory bulbectomy (OB) rats, which are commonly used as a model of depression, displayed decreased expression of NR1 in the hippocampus [10]. However, the representative antidepressant fluoxetine increased the expression of NR1 subunits at synaptic membranes and significantly reduced the levels of anxiety and learned helplessness in rats [11]. All of these results support the notion that the dysregulation of the NR1 subunit may contribute to the pathophysiology of affective disorders and the mechanism of antidepressant treatment. We therefore assessed the phosphorylation of NR1 in the hippocampus of CCI rats and investigated the possible effects of EA treatment on the NR1 subunit.

In the present study, continuous EA treatment completely abolished the CCI-induced anxiety-like behaviors and partially improved the depression-like behaviors in CCI rats. Moreover, a decrease in NR1 phosphorylation was observed in the hippocampus of rats subjected to CCI, and chronic EA treatment effectively reversed the decline of pNR1. All these results may provide new experimental basis for clinical EA application in treating chronic-pain-induced emotional disorders.

## 2. Materials and Methods

### 2.1. Animals

Experiments were performed on adult (180–200 g) male Sprague Dawley rats. Animals were obtained from the Experimental Animal Center, Shanghai Institutes for Biological Science, Chinese Academy of Sciences. They were housed under a 12/12 hour light/dark cycle at a room temperature of 23 ± 1°C with food and water ad libitum. All experiments were conducted in accordance with the National Institutes of Health Guide for the Care and Use of Laboratory Animals and the Ethical Issues of the International Association for the Study of Pain (IASP) [12].

### 2.2. CCI Neuropathic Pain Model

The rats were anesthetized with 1% pentobarbital sodium (i.p. 40 mg/kg), and the skin around the incision was disinfected with 75% ethanol. Then, the surgery was carried out according to the method of Bennett and Xie [13]. Briefly, the right common sciatic nerve was exposed by blunt dissection, and four ligatures (4-0 chromic catgut) were tied loosely around the sciatic nerve. Finally, muscle and skin were sutured in layers with silk suture. “Sham surgery” refers to the procedure of exposing the nerve as above without nerve ligation.

### 2.3. Von Frey Test for Mechanical Allodynia

Mechanical allodynia was measured using a series of von Frey filaments according to the method described by Dixon [14]. Before the test, the rats were placed in the mesh-floored plastic cage to acclimate for at least 30 minutes. Then, an ascending series of von Frey filaments with logarithmically incremental stiffness (0.40, 0.60, 1.4, 2.0, 4.0, 6.0, 8.0, and 15.0 g) (Stoelting, Wood Dale, IL, USA) was used during the test. The mid-plantar surface of each hind paw was stimulated perpendicularly in the experiment. The testing contained five more stimuli after the first change in response occurred, and the final score was converted to a 50% von Frey threshold using the method described by Dixon.

### 2.4. Hargreaves' Test for Thermal Hyperalgesia

Thermal hyperalgesia was measured with an IITC Model 390 Paw Stimulator Analgesia Meter (Life Science Instruments, Woodland Hills, CA). The rats were placed separately in clear plastic cages floored with window glass for an adaptation period of 30 min, and then radiant heat was applied to the plantar surface of the paw until the animal lifted its paw from the floor. The paw withdrawal latency (PWL), defined as the time from onset of radiant heat application to withdrawal of the rat's hind paw, was recorded per a previously described method [15].

### 2.5. Forced Swimming Test (FST)

The FST was conducted at the same time of day (9:00–16:00) using the method described by Porsolt et al. [16]. During the test, the rats were individually put into a glass cylinder (40 cm  height × 18 cm  diameter) containing 30 cm of water at 23 ± 2°C. The test consisted of two sections: first, a 15-minute training test; 24 hours later, the rats were subjected to a 5-minute test section. Within this section, the durations of climbing (upward-directed movements of the forepaws) and immobility (floating passively or making slight movements to keep the head above the water line) behavior were monitored and recorded. The water was changed between rats.

### 2.6. Elevated Plus Maze (EPM)

The EPM test was performed according to the method described previously [17]. The apparatus used for the elevated plus maze test was placed in a quiet room; the two open arms (50 cm × 10 cm) were perpendicular to two closed arms of equal size. The percentage of open arm entries and the percentage of time spent in the open arms were measured during a 5-min testing period by an automatic system (RD1108-EPM-R, Shanghai Mobiledatum Corporation, Shanghai, China).

### 2.7. Drug Administration

Fluoxetine hydrochloride (Eli Lilly and Company, Indianapolis, Indiana, USA) was suspended in 0.9% saline and administered once daily from day 7 to day 21 after the CCI surgery. The drug was administered through the orogastric gavage route with a single injection volume of fluoxetine (10 mg/kg and 30 mg/kg in 3 mL).

### 2.8. Electroacupuncture (EA)

EA treatment was applied to rats every other day from day 7 to day 21 after the CCI surgery. During the EA treatment, the trunk of the rat was kept immobile while the head and 4 limbs were kept free to move in a specially designed holder. A pair of stainless steel needles of 0.3-mm diameter were inserted with depths of 3 mm and 6 mm, respectively, into the acupoints “Bai-Hui” (GV20, located above the apex auriculate, on the midline of the head) and contralateral “Yang-Ling-Quan” (GB34, located near the knee joint, anterior and inferior to the small head of the fibula, in muscle peroneus longus and brevis). The two needles were connected with the output terminals of an EA apparatus (Model LH202H, Beijing Huawei Medical Instrument, P.R. China). Alternating strings of dense-sparse frequencies (100 Hz for 1.05 s and 2 Hz for 2.85 s, alternately) were selected. The intensity was adjusted to induce slight muscle contraction of the hind limb (≤3 mA). In the sham EA group, the needles were applied to the rats on the same acupoints as description above but without any electrical stimulation.

### 2.9. Western Blot

The hippocampi of the rats were quickly removed and ultrasonically disrupted in RIPA buffer (50 mM Tris (pH 7.4), 150 mM NaCl, 1% Triton X-100, 1% sodium deoxycholate, 0.1% sodium dodecyl sulfonate, sodium orthovanadate, sodium fluoride, ethylene diamine tetraacetic acid, and leupeptin), followed by centrifugation at 12000 ×g. The total protein level in the supernatants was measured using the Pierce BCA Protein Assay Kit (Thermo Scientific, Rockford, IL, USA). Samples were separated on 8% acrylamide gels and then transferred onto polyvinylidene fluoride membranes. After blocking with 5% nonfat milk in tris-buffered-saline with tween (TBST) (20 mM Tris-HCl, pH 7.5, 150 mM NaCl, and 0.05% Tween-20) for 1 hour at room temperature, the membranes were incubated with the primary antibody, rabbit anti-pNR1 (ser897) (1 : 1000: Millipore Bedford, MA), rabbit anti-total-NR1 (1 : 1000: Millipore Bedford, MA), or horseradish peroxidase- (HRP-) mouse-anti-GAPDH (1 : 10000: KangChen, Shanghai, China) at 4°C overnight. Then, the blots were washed in TBST and incubated in the appropriate secondary antibody, HRP-goat-anti-rabbit (1 : 2000, Santa Cruz Biotechnology, Santa Cruz, CA, USA) for 1 hour at room temperature. Western blot images were captured on an ImageQuant LAS4000 mini image analyzer (GE Healthcare, Buckinghamshire, UK), and the band levels were quantified using Image J software, version 1.42q.

### 2.10. Statistical Analysis

The data are presented as the mean ± standard error (SEM), and all statistical analyses were performed using Statistical Package for the Social Sciences (SPSS) 17.0 statistical software (SPSS Inc., Chicago, IL). The statistical significance of differences between groups was analyzed with Student's *t*-test or a one-way analysis of variance (ANOVA) followed by the Bonferroni posttest. In all statistical analyses, *P* < 0.05 was considered the threshold for statistical significance.

## 3. Results

### 3.1. CCI Induced Pain-Like Behaviors

**Figure 1:**
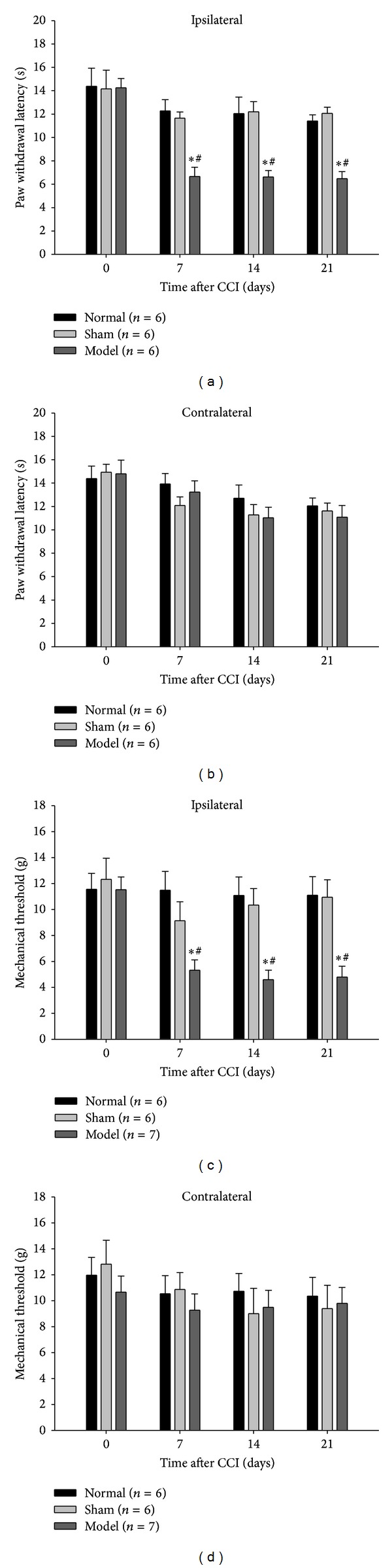
Chronic constriction injury (CCI) induced pain-like behavior. Thermal hyperalgesia and mechanical allodynia were measured before surgery, day 7, day 14, and day 21 after the CCI surgery, respectively. (a, b) Compared with the normal rats and sham controls, the rats subjected to CCI displayed significantly decreased PWL in the ipsilateral hind paw at 7, 14, and 21 days following the CCI surgery, but showed no reduction in the contralateral PWL at the same time point. (c, d) The mechanical threshold of CCI rats was statistically decreased in the ipsilateral hind paw at 7, 14, and 21 days after the surgery. There was no significant difference in either thermal or mechanical pain behaviors between the normal rats and sham controls. The data are expressed as the mean ± SEM. (*P < 0.05 versus normal group. ^#^ *P* < 0.05 versus sham surgery group).

In the Hargreaves test, CCI on rats' right hind limb provoked a significant decrease in the ipsilateral PWL beginning on day 7 after the sciatic nerve injury and stably sustained for three weeks (Figures 1(a) and 1(b)). And it also induced a profound decrease in the mechanical threshold of the ipsilateral hind paw over the same timeframe (Figures 1(c) and 1(d)). There was no significant difference in these pain-related behaviors between normal and sham surgery rats. These data suggested that the rats subjected to CCI developed obvious thermal hyperalgesia and mechanical allodynia.

### 3.2. CCI Induced Depression and Anxiety-Like Behaviors

**Figure 2:**
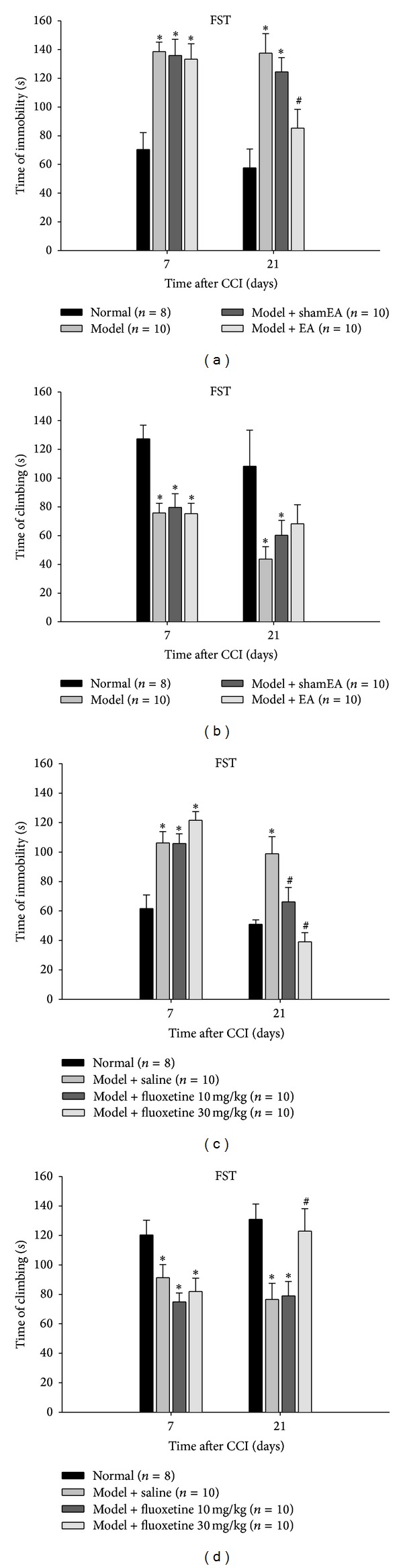
Effects of EA and fluoxetine on depression-like behaviors in CCI rats. Comparing with the normal rats, the rats subjected to CCI displayed a significant increase in the immobile time and a reduction in the climbing time at day 7 and day 21 after the CCI surgery. (a, b) Chronic EA at GV20–GB34 significantly shorten the immobile time of CCI rats but had no effect on the time of climbing behaviors. The data are expressed as the mean ± SEM. (*P < 0.05 versus normal rats. ^#^ *P* < 0.05 versus model group). (c, d) Chronic fluoxetine administration at both 10 and 30 mg/kg statistically reduced the immobile behaviors of CCI rats, respectively. Only the treatment of fluoxetine at 30 mg/kg increased the time spent in climbing behaviors. The data are expressed as the mean ± SEM. (*P < 0.05 versus normal rats. ^#^ *P* < 0.05 versus model + saline group).

**Figure 3:**
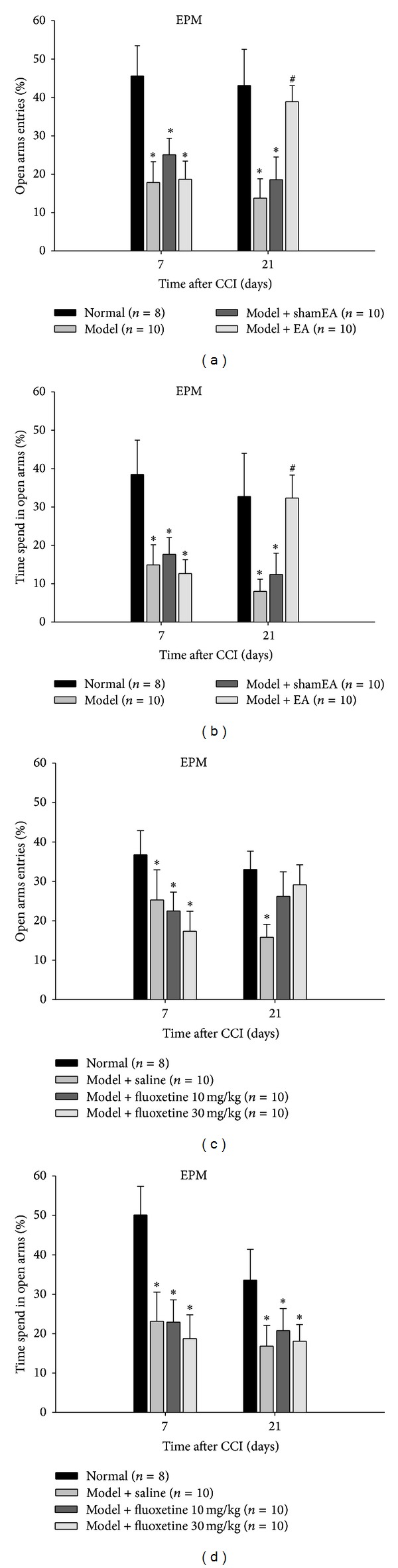
Effects of EA and fluoxetine on the anxiety-like behaviors in CCI rats. Comparing with the normal rats, the rats subjected to CCI displayed a significant decrease in the percentage of open arm entries and the percentage of time spent in open arms at day 7 and day 21 after the CCI surgery. (a, b) Chronic EA at GV20–GB34 on CCI rats significantly elevated their percentage of open arm entries and the percentage of time spent in open arms. The data are expressed as the mean ± SEM. (*P < 0.05 versus normal rats. ^#^ *P* < 0.05 versus model group). (c, d) Chronic fluoxetine at either 10 or 30 mg/kg induced no statistically therapeutic effects on the behaviors of CCI rats in the EPM test. The data are expressed as the mean ± SEM. (*P < 0.05 versus normal rats. ^#^ *P* < 0.05 versus model + saline group).

To investigate whether rats subjected to the CCI surgery developed depression- and anxiety-like behaviors, the FST and EPM tests were performed on day 7 and day 21, respectively. During the FST, CCI induced a marked prolongation of the immobile time as well as an obvious reduction in the climbing time (Figure 2). In the EPM experiment, the percentage of open arm entries, and the percentage of time spent in the open arms were all significantly decreased in the CCI group compared with the normal and sham surgery groups as controls (Figure 3). There was no significant difference in these emotion-related behaviors between normal and sham surgery rats.

Additionally, the open field test (OFT) was carried out to exclude the possibility that the lesion of the CCI surgery affects the performance of rats displayed in the FST and EPM tests. In the OFT, CCI rats displayed no reduction in the horizontal or vertical movements (data not shown), which proved that the locomotor activity of CCI rats was not impaired by the surgery. Collectively, these results suggested that rats subjected to the CCI neuropathic pain developed significant depression- and anxiety-like behaviors.

### 3.3. Effects of EA at GV20–GB34 on the Depression-Like Behaviors in CCI Rats

To evaluate the curative effect of EA treatment on the chronic-pain-induced emotional behaviors, the classic antidepressant fluoxetine was applied to another group of CCI rats beginning on day 7 after the surgery as comparison. In the FST, chronic EA treatment partially reversed the CCI-induced depression-like behaviors. EA at GV20–GB34 caused a significant reduction in the immobile time of CCI rats, but had no influence on the climbing time (Figures 2(a) and 2(b)). Correspondingly, fluoxetine at the dose of 30 mg/kg for 14 consecutive days not only reversed the CCI-induced prolongation of the immobility time (Figure 2(c)) but also extended the climbing time of CCI rats (Figure 2(d)).

### 3.4. Effects of EA at GV20–GB34 on the Anxiety-Like Behaviors in CCI Rats

In the EPM test, EA treatment for continuous 2 weeks significantly improved the anxiety-like behaviors of CCI rats, characterized by an obvious increase in the percentage of open arm entries and the percentage of time spent in the open arms (Figures 3(a) and 3(b)). However, chronic fluoxetine at either 10 mg/kg or 30 mg/kg produced no significant effect on the anxious behaviors of CCI rats (Figures 3(c) and 3(d)).

These results revealed that continuous EA treatment markedly improved the CCI-induced behavioral deficits. And in the present study, EA had an advantage over the antidepressant fluoxetine in terms of the anxiolytic effect.

### 3.5. Effects of EA at GV20–GB34 on the Pain-Like Behaviors in CCI Rats

**Figure 4:**
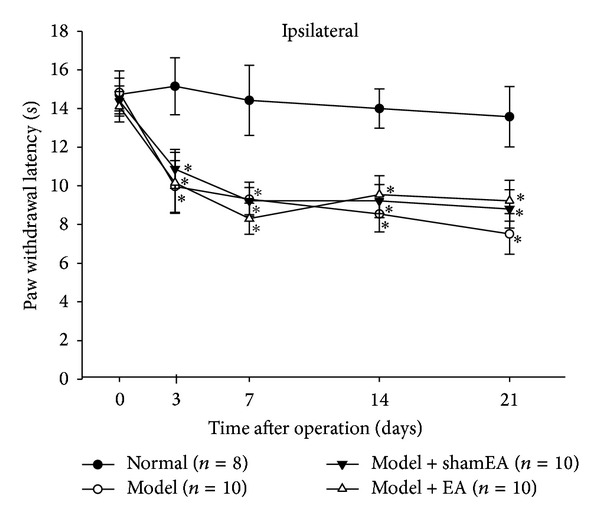
Effects of EA on the CCI-induced thermal hyperalgesia. Thermal hyperalgesia was measured before surgery, day 3, day 7, day 14, and day 21 after the CCI surgery, respectively. Compared with the normal rats, the ipsilateral PWL of CCI rats was significantly decreased from day 3 and sustained to day 21 after the CCI surgery (*P* < 0.05). EA treatments were applied to rats every other day from day 7 to day 21. Neither chronic EA nor sham EA at GV20–GB34 had effect on the ipsilateral PWL of CCI rats (*P* > 0.05). The data are expressed as the mean ± SEM. (*P < 0.05 versus normal rats).

The thermal hyperalgesia of rats receiving EA treatment was also monitored at day 3, day 7, day 14, and day 21 after the CCI surgery. Chronic EA at GV20–GB34 had no effect on CCI-induced thermal pain behaviors (Figure 4), which further illustrated that the therapeutic action of EA treatment on neuropathic-pain-induced emotional behavior was not due to an analgesic effect.

### 3.6. Effects of EA at GV20–GB34 on the Phosphorylation of Hippocampal NR1 Subunits in CCI Rats

**Figure 5:**
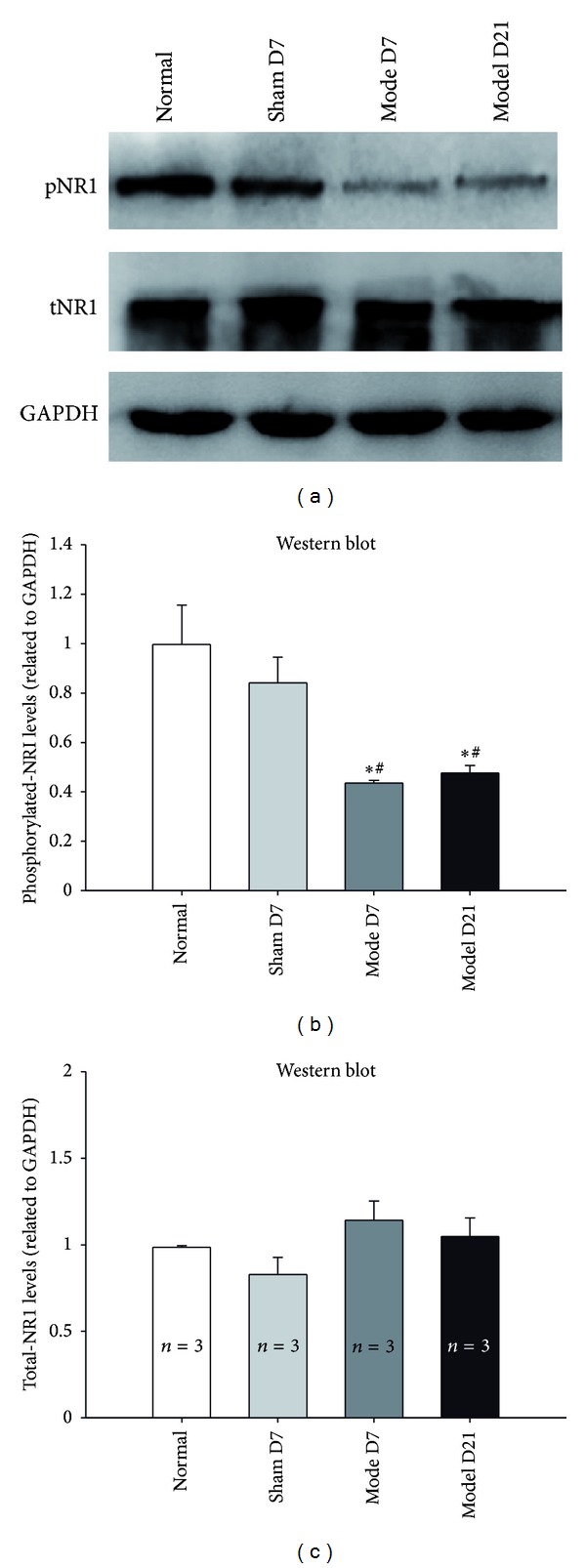
CCI reduced the phosphorylation of hippocampus NR1 subunits. (a) Representative western blots showing the expression of pNR1, total NR1, and GAPDH in contralateral hippocampus. (b) Compared with the normal group and sham controls, the phosphorylation of NR1 was significantly reduced in the hippocampus of CCI rats at day 7 and day 21 after the CCI surgery (normalized by GAPDH). (c) The expression of total NR1 was not decreased in the hippocampus of CCI rats at the same time point (*n* = 3). The data are expressed as the mean ± SEM. (*P < 0.05 versus normal group. ^#^ *P* < 0.05 versus sham surgery group).

**Figure 6:**
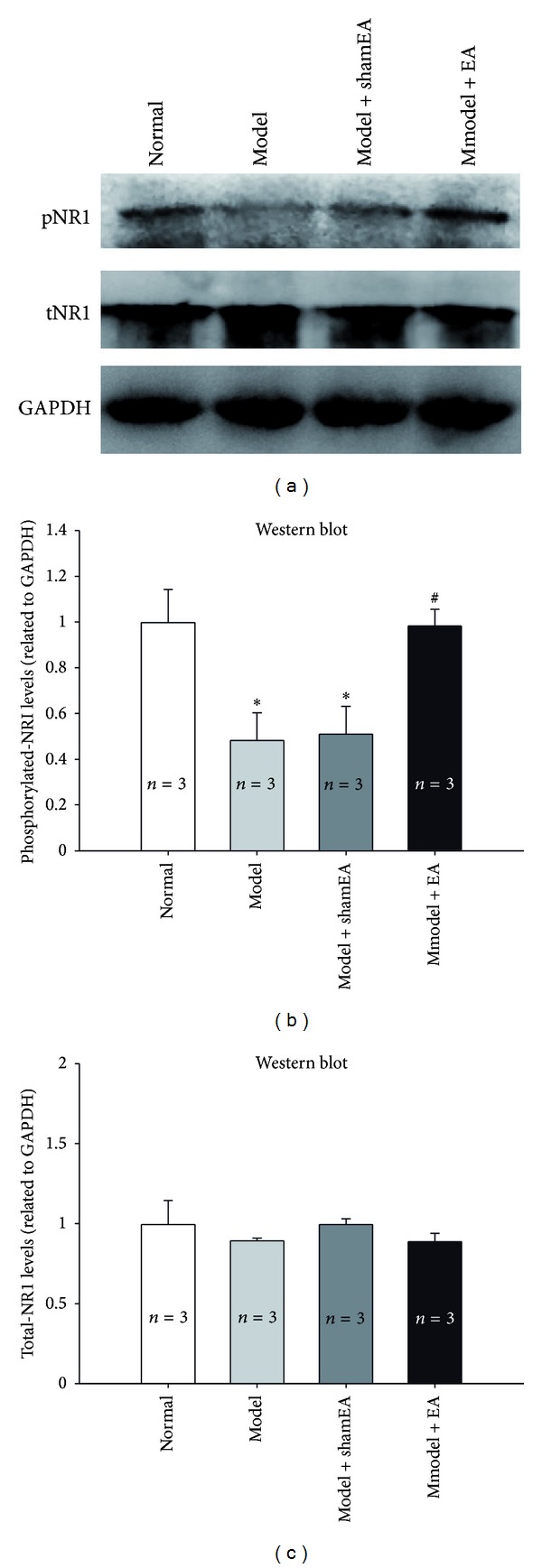
Effects of EA at GV20–GB34 on the phosphorylation of hippocampus NR1 subunits. (a) Representative western blots showing the expression of pNR1, total NR1, and GAPDH in contralateral hippocampus. (b) Chronic EA treatment at GV20–GB34 significantly reversed the CCI induced low level of pNR1 in the hippocampus, but the sham EA treatment had no effects on NR1 phosphorylation. (c) There was no significant difference in the expression of total NR1 among rats subjected to different treatments (*n* = 3). The data are expressed as the mean ± SEM. (*P < 0.05 versus normal group. ^#^ *P* < 0.05 versus model group as control).

Compared with the normal rats, the level of pNR1 was obviously decreased in the contralateral hippocampus of CCI rats at both day 7 and day 21 after the surgery, with no changes in the expression level of total NR1 (Figures 5(b) and 5(c)). EA at GV20–GB34 for 14 days significantly restored the decreased NR1 phosphorylation of CCI rats, but sham EA had no effects on the level of pNR1 (Figures 6(b) and 6(c)). These results suggested that the phosphorylation of NR1 in hippocampus may be associated with the therapeutic effect of EA.

## 4. Discussion

The present study evaluated the therapeutic effects of EA on neuropathic-pain-induced emotional disorders for the first time. After the CCI surgery, rats displayed significant depression- and anxiety-like behaviors. Continuous EA treatment not only blocked the CCI-induced behavioral deficits but also reversed the decrease in pNR1 in the hippocampus of CCI rats, suggesting a potential mechanism underlying the antidepressive and anxiolytic effects of EA treatment.

The psychiatric disorders induced by chronic neuropathic pain are significant clinical concerns. In several clinical studies, neuropathic-pain-associated depressive and anxiety symptoms were adequately described [18]. However, only a few animal studies have been conducted to mimic patients with pain-related emotional disorders and further investigate the underlying mechanisms [19, 20]. In the present study, the CCI model was used for studying the neuropathic-pain-induced emotional deficits because their significant and stable behaviors are induced by neuropathic pain [21].

As described in the previous literatures, the rats subjected to CCI surgery walked with a definite limp at the first few postoperative days. After a week or so the gait of the hind paw became very consistent [13]. The rats' potential impairments on locomotion activity may influence the results of FST and EPM, such as facilitating the immobile behaviors during the FST [16]. So it is extremely important to exclude this interference factor when presenting the results of FST and EPM. In the present study, the FST and EPM were performed on day 7 and day 21 after the CCI surgery. So we conducted the OFT on day 7 and day 21 accordingly. In the open field test, the total distance traveled and the vertical movement numbers were recorded. Our data presented that the CCI rats displayed no reduction in horizontal or vertical movements, which demonstrated the normal locomotion activity of CCI rats. Our conclusion was also in accordance with the findings described before [22, 23]. In conclusion, the OFT results verified that the significant differences in FST and EPM were resulted from CCI-induced emotional disorders rather than the surgery-induced defects in motor ability. All these results further confirmed that the rats subjected to CCI developed depression- and anxiety-like behaviors.

EA has been broadly accepted to alleviate pain when stimulating electric current is applied to acupoints with acupuncture needles [24, 25], and accumulating evidence also shows that EA has been widely applied to clinical patients with depression diseases [26, 27]. First, we evaluate the treatment effect of EA on the psychiatric disorders induced by chronic neuropathic pain. The acupoints chosen in this study have been primarily used clinically for treating patients with mental diseases, which may be why EA at GV20–GB34 effectively improved only the emotional behaviors and not the pain behaviors of CCI rats (Figure 3). Additionally, to better assess the antidepressive and anxiolytic effects of EA treatment, we also administered fluoxetine, a representative member of the selective serotonin reuptake inhibitor (SSRI) class of antidepressants [28]. Interestingly, EA displayed obvious advantages over the fluoxetine in the treatment of CCI-induced anxiety in the EPM test (Figure 2), providing a new insight into the clinical application of EA.

The exact mechanism by which EA exerts its antidepressive and anxiolytic effects remains unknown, but the hippocampus pNR1 modulation during the EA treatment may provide some suggestions. As previously reported, NR1 phosphorylation at S897 by protein kinase A reduced the endoplasmic reticulum (ER) retention and facilitated NR1 exiting from the ER to the cell surface [29, 30], and the decreased phosphorylation of NR1 at S897 was proved to be related to multiple psychiatric disorders and cerebral diseases, including schizophrenia and NMDA-induced brain damage [31–33]. Thus, the CCI-induced reduction of hippocampus pNR1 might downregulate the translocation of NR1 subunits and consequently impair NR1-mediated neural activity. In the present study, we described the effects of EA treatment on the phosphorylation of NR1 at Ser897, suggesting that the modulation of pNR1 might be associated with the therapeutic effects of EA treatment on CCI-induced emotional disorders.

In summary, the rats with neuropathic pain developed depression- and anxiety-like behaviors. Furthermore, chronic EA has remarkable therapeutic effects on CCI-related emotional disorders. The therapeutic effects of EA treatment might be correlated with its regulatory actions on hippocampal pNR1. The present study suggested an effective alternative treatment for chronic-pain-induced psychiatric deficits.
